# Associated costs of hospitalizations due to external causes: time series analysis, Brazil, 2000-2023

**DOI:** 10.1590/S2237-96222026v35e20240889.en

**Published:** 2026-04-10

**Authors:** Araceli Moreira De Martini Fontenele, Alcione Miranda dos Santos, Luz Marina Gómez Gómez, Fábio Nogueira da Silva, Bruno Luciano Carneiro Alves de Oliveira

**Affiliations:** 1 Universidade Federal do Maranhão, Departamento de Saúde Pública, São Luís, MA, Brazil

**Keywords:** Accidents, Violence, Morbidity, Hospital Costs, Time Series Studies, Accidentes, Violencia, Morbilidad, Costos de Hospital, Estudios de Series Temporales

## Abstract

**Objective:**

To analyze the temporal trend in case fatality and mean length of hospital stay, as well as the costs of hospitalizations due to external causes in Brazil between 2000 and 2023.

**Methods:**

Time series study using data on hospitalizations due to external causes recorded in the Hospital Information System (SIH). Temporal trend analysis of mean length of stay and case fatality was performed using joinpoint regression, with calculation of the annual percentage change (APC), average annual percentage change (AAPC), and 95% confidence intervals (95%CI). Costs were measured using macro-costing.

**Results:**

From 2000 to 2023, 23,009,176 (8.1%) hospitalizations due to external causes were recorded. Most occurred in the Southeast region (40.2%), among men aged 20–39 years (40.4%) and older women (31.4%). Falls were the main cause (39.8%). During this period, the mean length of stay decreased across all age groups, with the greatest reduction in the 0–9-year age group (AAPC −1.27; 95%CI −1.43; −1.13) and the smallest among older adults (AAPC −0.51; 95%CI −0.77; −0.29). There was an increasing trend in length of stay among older men between 2003 and 2017 (APC 0.41; 95%CI 0.15; 2.46) and among older women between 2005 and 2017 (APC 0.72; 95%CI 0.30; 2.49). Case fatality showed a decreasing trend between 2000 and 2023 (AAPC −0.58; 95%CI −1.10; −0.04), with higher rates among older adults (men: 7.2%; women: 5.0%). Hospitalizations incurred costs totaling BRL 44.24 billion, with higher expenditures in the Southeast region (BRL 18.33 billion) and among men (BRL 30.65 billion).

**Conclusion:**

External causes remain a serious public health problem in Brazil. Despite the reduction in case fatality, their costs remain high, requiring strengthening of the health care network and control of the determinants of these health conditions.

## Resumen

**Objetivo:**

Analizar la tendencia temporal de la letalidad y de la estancia hospitalaria media, así como los costos de las hospitalizaciones por causas externas en Brasil entre 2000 y 2023.

**Métodos:**

Estudio de series temporales con datos de hospitalizaciones por causas externas registrados en el Sistema de Información Hospitalaria (SIH). El análisis de la tendencia temporal de la estancia media y de la letalidad se realizó mediante regresión por puntos de inflexión, con cálculo de la variación porcentual anual (VPA), de la variación media del período (VMP) y de los intervalos de confianza del 95% (IC95%). Los costos se estimaron mediante el macrocosting.

**Resultados:**

Entre 2000 y 2023, se registraron 23.009.176 hospitalizaciones (8,1%) por causas externas. La mayoría ocurrió en la región Sudeste (40,2%), entre hombres de 20–39 años (40,4%) y mujeres adultas mayores (31,4%). Las caídas fueron la principal causa (39,8%). En el período, la estancia hospitalaria media mostró una tendencia decreciente en todos los grupos etarios, con la mayor reducción en el grupo de 0–9 años (VMP −1,27; IC95% −1,43; −1,13) y la menor en el grupo de personas mayores (VMP −0,51; IC95% −0,77; −0,29). Se observó una tendencia creciente de la estancia en hombres mayores entre 2003 y 2017 (VPA 0,41; IC95% 0,15; 2,46) y en mujeres mayores entre 2005 y 2017 (VPA 0,72; IC95% 0,30; 2,49). La letalidad presentó una tendencia decreciente entre 2000 y 2023 (VMP −0,58; IC95% −1,10; −0,04), con mayores proporciones en personas mayores (hombres: 7,2%; mujeres: 5,0%). Las hospitalizaciones generaron un costo total de R$ 44,24 mil millones, con mayores gastos en la región Sudeste (R$ 18,33 mil millones) y entre los hombres (R$ 30,65 mil millones).

**Conclusión:**

Las causas externas continúan siendo un grave problema de salud en Brasil. A pesar de la reducción de la letalidad, los costos siguen siendo elevados, lo que exige fortalecer la red de atención en salud y controlar los determinantes de estos eventos.

**Ethical aspects**

This research used public domain anonymized databases.:

## Introduction 

External causes are among the main global public health problems and the leading causes of premature and avoidable deaths, accounting for nearly five million (9%) deaths annually worldwide (1). In Brazil, they constitute the fourth leading group of causes of death, after cardiovascular diseases, respiratory tract diseases, and neoplasms, and predominantly affect men and young people (1,2). Arising from multiple inequities, external causes contribute to a high burden of hospitalizations, elevated health care and social security costs, disabilities, and loss of productivity among affected individuals (1,3).

Hospitalizations due to external causes account for 6% of Brazil’s gross domestic product (3) and have increased over the years. In 1998, the hospitalization rate in public hospitals affiliated with the Brazilian Unified Health System (*Sistema Único de Saúde*, SUS) was 37.1 cases per 10 thousand inhabitants. In 2010, the rate increased to 48.7 per 10 thousand inhabitants (a 31.3% increase). In 2021, the rate reached 58.4 per 10 thousand inhabitants, representing an increase of nearly 20% over the last decade (4,5). In the same year, nearly 1.25 million people were hospitalized in Brazil due to external causes, with a mean length of stay of 4.7 days and a total cost (BRL 1.65 billion) six times higher than that recorded in 2011 (1).

These health conditions affect the entire health system, generating a high number of complex cases that add to those resulting from other health conditions, necessitating the reorganization of services, human and financial resources, and equipment (6). Although mortality data are more frequently analyzed, it is estimated that for each death due to injury, there are 30 victims hospitalized due to external causes, suggesting a diversity in the morbidity profile compared with that of mortality (7).

Studies report an increase in the incidence of conditions due to external causes (8,9). However, there is limited understanding of the distribution, trends, and socioeconomic implications of these hospitalizations. External causes exhibit a heterogeneous distribution across individual and contextual characteristics. Evidence indicates that characteristics of hospitalization cases are associated with in-hospital mortality, and that the care provided also determines length of stay (10,11). Understanding the epidemiological characteristics of morbidity from external causes can inform the planning of health surveillance actions and the monitoring of public policies. 

This study aimed to analyze the temporal trend in case fatality and mean length of hospital stay, as well as the costs of hospitalizations due to external causes in Brazil between 2000 and 2023. 

## Methods 

### Study design

This is a time series analysis using data on hospitalizations due to external causes recorded in the Hospital Information System of the Unified Health System of the Brazilian Unified Health System (*Sistema de Informações Hospitalares do SUS*, SIH/SUS), managed by the Brazilian Unified Health System Information Technology Department (*Departamento de Informação e Informática do Sistema Único de Saúde*, DATASUS). Records of Hospital Admission Authorizations (*Autorizações de Internação Hospitalar*, AIH) occurring between 2000 and 2023 were selected.

### Setting 

Brazil, located in South America, has an area of 8,510,417.77 km^2^ and an estimated population of 203 million inhabitants. The country, composed of 27 federative units (26 states and the Federal District), distributed across five regions—North, Northeast, Southeast, Central-West, and South—faces health challenges related to hospitalizations for external causes, which pose a significant socioeconomic problem. Hospitalizations due to these conditions reveal their dynamics and associated costs (12).

### Participants 

There are two approaches to obtaining estimates from DATASUS and classifying external causes: conservative and non-conservative (12). In the conservative approach, hospitalizations are identified in two ways: (1) when codes from Chapter XX of the International Statistical Classification of Diseases and Related Health Problems 10th Revision (ICD-10) (V01–Y98) appear in the primary diagnosis and any other code appears in the secondary diagnosis; (2) when codes from Chapter XX of ICD-10 appear in the secondary diagnosis, the primary diagnosis must contain only codes from Chapter XIX of ICD-10 (S00–T98), which refer to injuries, poisoning, and certain other consequences of external causes (13). In the non-conservative approach, hospitalizations are counted based on the presence of an ICD-10 code, regardless of whether it appears in the primary or secondary diagnosis. In the SIH database, in the row corresponding to each record and in the column for the primary or secondary diagnosis variable, a code referring to Chapter XX must be present (13). 

In this study, AIH records were selected by hospitalization year and identified using a non-conservative approach (13). This approach identified more cases than the conservative approach and provided a clearer view of the phenomenon’s size as a primary cause of hospitalization. In the conservative approach, if Chapter XIX of ICD-10 is required for the primary diagnosis, some cases may be excluded, whereas this does not occur with the non-conservative approach. The possibility of identifying records that had been coded inconsistently with ICD-10 or SIH-SUS but represented hospitalizations due to external causes was also considered (14,15). 

### Variables 

The following variables were used: (i) sociodemographic variables: age group (in years): 0–9; 10–19; 20–39; 40–59; ≥60; sex (male; female); region of Brazil (North; Northeast; Central-West; Southeast; South); location (capital city; non-capital municipalities); (ii) groups of external causes: transport accidents (V01–V99); assaults (X85–Y09); falls (W00–W19); intentional self-harm (X60–X84); events of undetermined intent (Y10–Y34); other external causes (W20–X59); (iii) indicators: proportion of hospitalizations due to external causes; rate of hospitalizations due to external causes paid for by the SUS; mean length of stay (in days); proportion of in-hospital case fatality (deaths); total value of hospitalizations paid for (costs in Brazilian reais).

### Data sources and measurement

The data source was the AIH database of the SIH-SUS (reduced RD-AIH database), managed by DATASUS. Non-identified microdata were used, obtained via the microdatasus package in R (16). Initially, the devtools package was installed in R. Subsequently, the microdatasus package (GitHub repository: https://github.com/) was installed using the code: devtools::install_github (“rfsaldanha/microdatasus”). Finally, the fetch_datasus function was used to access, download, and read the microdata (16). 

Data extraction was performed by year (2000–2023). As of 2014, the SIH database structure was modified to include nine additional variables for recording secondary diagnoses: DIAGSEC1 to DIAGSEC9 (13). The fields previously designated for recording this variable began to be filled with zeros. To obtain hospitalization records without losses, two databases were created (an aggregated database from 2000 to 2014, which included the variable DIAG_SECUN under the previous classification, and another from 2015 to 2023, with the variables DIAGSEC1 to DIAGSEC9 under the current classification). 

In this study, to analyze the completeness of variables (Supplementary Table 1), criteria of completeness, consistency, and reliability were applied (17). Completeness was classified as: good (≥75.1%); regular (50.1% to 75.0%); low (25.1% to 50.0%); very low (≤25.0%). Consistency was classified as excellent (≥90%), regular (70%–89%), or low (<70%). Variables with low consistency percentages were not considered. Given its importance for identifying and characterizing external causes, only the secondary diagnosis variable was retained, despite its previously documented incomplete recording. Reliability was based on meeting the criteria for completeness (>50.1%) and consistency (>70%). After this analysis, the databases were aggregated. An updated database as of 15/9/2024 was used. Filters were applied for the period 2000–2023 and for external causes. 

**Table 1:** Absolute frequencies (n) and relative frequencies (%) of hospitalizations due to external causes funded by the Brazilian Unified Health System (SUS), according to sociodemographic characteristics and cause groups, by sex. Brazil, 2000–2023 (n=23,009,176)

Characteristics	Total	Male	Female
	n (%)	n (%)	n (%)
**Age group** (years)			
0–9	2,127,047 (9.2)	1,344,092 (8.5)	782,955 (10.9)
10–19	3,012,101 (13.1)	2,314,262 (14.6)	697,839 (9.8)
20–39	8,182,711 (35.6)	6,408,019 (40.4)	1,774,692 (24.8)
40–59	5,534,824 (24.1)	3,881,163 (24.5)	1,653,661 (23.1)
≥60	4,152,493 (18.0)	1,906,937 (12.0)	2,245,556 (31.4)
**Region of the country**			
North	2,098,646 (9.1)	1,503,814 (9.5)	594,832 (8.3)
Northeast	5,688,721 (24.7)	4,013,906 (25.3)	1,674,815 (23.4)
Central-West	2,211,728 (9.6)	1,540,873 (9.7)	670,855 (9.4)
Southeast	9,244,974 (40.2)	6,261,088 (39.5)	2,983,886 (41.7)
South	3,765,107 (16.4)	2,534,792 (16.0)	1,230,315 (17.2)
Location			
Capital	5,212,116 (22.7)	3,538,352 (22.3)	1,673,764 (23.4)
Non-capital municipalities	17,797,060 (77.3)	12,316,121 (77.7)	5,480,939 (76.6)
**Cause groups**			
Transport accidents	4,217,581 (18.4)	3,259,714 (20.6)	957,867 (13.4)
Assaults	1,153,999 (5.0)	954,786 (6.0)	199,213 (2.8)
Falls	9,153,177 (39.8)	5,851,763 (36.9)	3,301,414 (46.1)
Voluntarily self-inflicted injuries	243,901 (1.0)	141,414 (0.9)	102,487 (1.4)
Events of undetermined intent^a^	1,691,063 (7.3)	1,163,413 (7.3)	527,650 (7.4)
Other external causes^b^	6,549,455 (28.5)	4,483,383 (28.3)	2,066,072 (28.9)

^a^Events of undetermined intent include injuries for which the available information does not allow distinction between accident, self-inflicted injury, and assault; ^b^Other external causes include exposure to mechanical forces, accidental drowning and submersion, other accidental risks to respiration, burns, and poisonings (accidental intoxication).

### Bias 

AIH records of long-term hospitalizations (AIH type 5: hospital stays longer than 30 days) were excluded from the analysis. External causes resulting from specific causes were not included: complications of medical and surgical care (ICD Y40–Y84); sequelae of external causes of morbidity and mortality (ICD Y85–Y89); and supplementary factors related to causes of morbidity and mortality classified elsewhere (ICD Y90–Y98). This choice was made because of the risk of duplicate records: individuals may have had prior hospitalizations for the aforementioned causes, which would not constitute external causes as the primary event leading to hospitalization. 

### Statistical methods

Initially, a descriptive analysis was performed. Subsequently, four indicators were estimated: (1) proportion of hospitalizations due to external causes (number of hospitalizations due to external causes according to place of residence/number of hospitalizations for all causes × 100); (2) hospitalization rate due to external causes paid by the SUS (number of hospitalizations due to external causes according to place of residence/resident population × 100.000 inhabitants); (3) mean length of stay (total days of hospitalization for external causes/total hospitalizations due to external causes during the period); (4) in-hospital case fatality proportion (hospitalizations due to external causes ending in death/total hospitalizations due to external causes during the period × 100). The calculation of the proportion and hospitalization rate was performed by sex. For the remaining indicators, sex, age group, and external cause group were considered. Hospitalization outcomes were discharge and death.

In calculating the hospitalization rate, census data and population estimates for each region, based on the 2010 demographic census from the Brazilian Institute of Geography and Statistics (*Instituto Brasileiro de Geografia e Estatística*, IBGE), were used, as provided by DATASUS (12). 

Temporal trend analysis was performed for the indicators mean length of stay and in-hospital case fatality, stratified by sex, age group, and external cause group. The study data were imported into the Joinpoint Regression Program, version 5.2.2.0, for segmented linear analysis of the time series (joinpoint regression) (18).

The Joinpoint Regression Program estimated the annual percent change (APC) and average annual percent change (AAPC). Temporal trends were classified as stationary (p-value>0.05), increasing (p-value<0.05 and positive regression coefficient), or decreasing (p-value<0.05 and negative regression coefficient). For all temporal trend analyses, 95% confidence intervals (95%CI) were used (18).

The macro-costing method was used in cost analyses (19). This method considers the total value of AIH for external causes paid during the analysis period, hospital length of stay, and expenses associated with hospital services. It is based on the *Extended National Consumer Price Index* (*Índice Nacional de Preços do Consumidor Amplo*, IPCA), considering annual inflation rates and values updated to the reference year, 2023. 

The macro-costing calculation represents the total number of hospitalizations and the total amounts spent by the SUS in the SIH (total cost) during the period from 2000 to 2023. The average cost per hospitalization was calculated by dividing the total cost of hospitalizations due to external causes by the number of approved (paid) AIH for these hospitalizations during the period. The mean total cost was calculated by dividing the total cost by the number of years in the analyzed period (24 years: 2000 to 2023). The costs measured refer exclusively to acute hospitalizations. Discharge and death were considered hospitalization outcomes. 

Analyses were performed using RStudio software, version 2024.4.4.0 (R Foundation for Statistical Computing, Boston, United States). 

## Results 

From 2000 to 2023, 283,149,255 hospitalizations were recorded in the Hospital Information System of the Unified Health System of the Brazilian Unified Health System (SIH/SUS). Of these, 23,009,176 (8.1%) were due to external causes. Hospitalizations predominated among individuals aged 20–39 years (35.6%) and 40–59 years (24.1%), in the Southeast (40.2%) and Northeast (24.7%) regions, and in other municipalities (77.3%). Significant differences were observed across all variables by patient sex. Among men, hospitalizations predominated in the 20–39-year age group (40.4%) and the 40–59-year age group (24.5%). Among women, they were higher in older women (≥60 years) (31.4%). Hospitalizations were most frequent in two regions for both sexes: men—39.5% in the Southeast and 25.3% in the Northeast; women—41.7% and 23.4%, respectively. Falls remained the most frequent cause (39.8%) within the cause groups, followed by other external causes (28.5%) (Table 1). 

**Figure 1:**
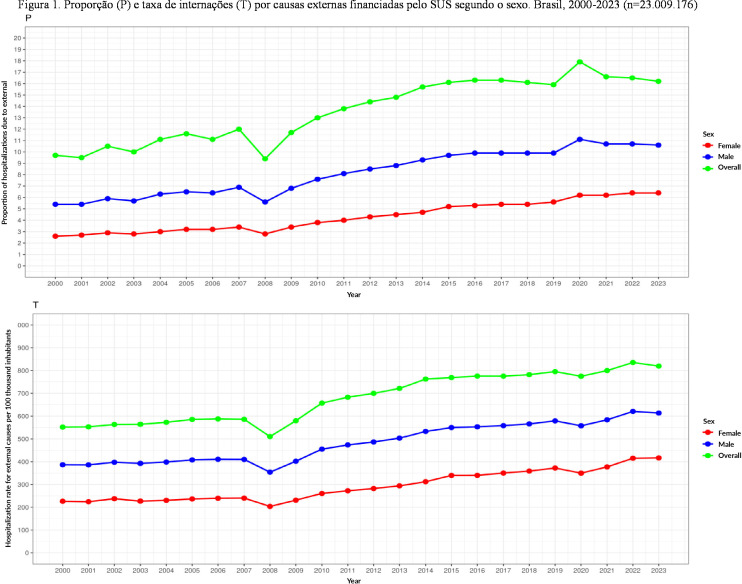
Proportion (P) and hospitalization rate (R) for external causes funded by the Brazilian Unified Health System (SUS), by sex. Brazil, 2000–2023 (n=23,009,176)

During the study period (2000–2023), the proportion of hospitalizations for external causes among men increased from 5.4% to 10.6%, representing a 96.3% increase. Among women, it rose by 146.1%, from 2.6% to 6.4%. The hospitalization rate due to external causes increased by 58.8% among men, from 386.5 to 613.7 hospitalizations per 100 thousand inhabitants. Among women, the increase was 84.3%, from 226.2 to 416.8 per 100 thousand inhabitants (Figure 1). 

A decreasing trend in mean hospital length of stay was observed across all age groups. The greatest reduction occurred in children aged 0–9 years (AAPC -1.27; 95%CI -1.43; −1.13) and the smallest among older adults (AAPC -0.51; 95%CI -0.77; -0.29). The highest mean lengths of stay were observed in older adults, 6.6 days for men and 6.3 days for women. Among men, an increasing trend was observed in adolescents from 2004 to 2016 (APC 0.80; 95%CI 0.47; 1.63) and in older adults from 2003 to 2017 (APC 0.41; 95%CI 0.15; 2.46). Among women, the trend was increasing in adults aged 20–39 years between 2005 and 2013 (APC 1.19; 95%CI 0.57; 3.08) and in those aged 40–59 years from 2004 to 2015 (APC 1.65; 95%CI 0.47; 8.10), as well as in older women from 2005 to 2017 (APC 0.72; 95%CI 0.30; 2.49) (Table 2). 

**Table 2:** Annual percent change (APC), average annual percent change (AAPC), 95% confidence intervals (95%CI) of in-hospital case fatality and mean length of stay, and total hospitalization costs for external causes funded by the Brazilian Unified Health System (SUS), by age group and sex. Brazil, 2000–2023 (n=23,009,176)

Characteristics		Length of stay					Case fatality			Total costs (BRL)		
Age group (years)	Sex	Mean^a^	Period	APV (95%CI)	Trend^b^	AAPC (95%IC)	Case fatality (%)^c^	Period	APV (95%CI)	Trend ^b^	AAPC (95%IC)	Adj. cost (BRL)^d^
0–9	Male	3.4	2000–2006	-2.10 (-3.82; -1.39)	↓	≥60 (-1.43; -1.13) ↓	0.6	2000–2023	-0.49 (-1.03; 0.06)	→	-0.30 (-0.92; 0.11) →	1.46 billion
			2006–2016	-0.41 (-0.74; 0.94)	→							
			2016–2023	-2.25 (-3.04; -1.73)	↓							
	Female	3.4	2000–2013	-1.17 (-1.76; -0.69)	↓		0.8	2000–2008	-2.01 (-10.21; 0.32)	→		885.57 million
			2013-2017	1.67 (-1.68; 3.54)	→			2008–2016	4.96 (2.60; 14.59)	↑		
			2017–2020	-5.27 (-6.76 ; 1.19)	→			2016–2023	-2.65 (-7.77; -0.27)	↓		
			2020–2023	0.03 (-2.68; 3.47)	→							
	Total	3.4					0.7					2.34 billion
10–19	Male	4.0	2000–2004	-1.95 (-4.66; -0.33)	↓	-0.85 (-0.99; -0.72) ↓	1.3	2000–2017	-1.17 (-1.75; -0.48)	↓	-2.90 (-3.39; -2.51) ↓	3.48 billion
								2017–2023	-7.73 (-11.42; -5.44)	↓		
			2004–2016	0.80 (0.47; 1.63)	↑							
			2016–2023	-3.30 (-4.07; -2.61)	↓							
	Female	3.9	2000–2016	-0.12 (-0.70; 2.97)	→		1.0	2000–2010	-1.23 (-2.51; 3.35)	→		991.33 million
			2016–2023	-2.21 (-7.01; -0.50)	↓			2010–2023	-4.19 (-6.55; -3.34)	↓		
	Total	4.0					1.2					4.47 billion
**20–39**	Male	4.9	2000–2005	-1.01 (-3.14; -0.04)	↓		2.1	2000–2004	0.03 (-2.32; 5.70)	→		12.49 billion
			2005–2015	0.42 (-0.10; 1.84)	→			2004–2023	-3.41 (-3.88; -3.17)	↓		
			2015–2023	-2.13 (-2.74; -1.55)	↓							
	Female	4.3	2000–2005	-1.55 (-4.20; -0.40)	↓	-0.80 (-0.97; -0.64) ↓	1.3	2000–2005	1.14 (-1.73; 9.27)	→	-2.90 (-3.17; -2.58) ↓	2.99 billion
			2005–2013	1.19 (0.57; 3.08)	↑			2005–2023	-3.73 (-4.50; -3.30)	↓		
			2013–2023	-1.92 (-2.41; -1.48)	↓							
	Total	4.8					1.9					15.48 billion
**40–59**	Male	5.5	2000–2023	-0.41 (-0.99; 0.16)	→	-0.82 (-1.00; -0.63) ↓	2.8	2000–2023	-2.29 (-2.69; -1.91)	↓	-2.48 (-3.41; -1.11) ↓	7.93 billion
	Female	4.7	2000–2004	-5.46 (-13.70; -1.02)	↓		1.8	2000–2021	-0.66 (-1.29; 2.53)	→		2.87 billion
			2004–2015	1.65 (0.47; 8.10)	↑			2021–2023	-15.0 (-25.39; -1.53)	↓		
			2015–2023	-2.85 (-8.09;-0.94)	↓							
	Total	5.3					2.5					10.80 billion
≥**60**	Male	6.6	2000–2003	-1.83 (-4.18; 0.09)	→	-0.51 (-0.77; -0.29) ↓	7.2	2000–2021	0.79 (0.45; 1.25)	↑	-0.02 (-0.59; 0.73) →	5.30 billion
			2003–2017	0.41 (0.15; 2.46)	↑			2021–2023	-11.91 (-16.88; -3.17)	↓		
			2017–2020	-4.19 (-5.71; 0.35)	→							
			2020–2023	0.13 (-2.44; 3.39)	→							
	Female	6.3	2000–2005	-0.95 (-3.48; 0.17)	→		5.0	2000–2013	1.52 (-3.43; 2.85)	→		5.86 billion
			2005–2017	0.72 (0.30; 2.49)	↑			2013–2016	9.57 (0.10; 13.56)	↑		
			2017–2020	-4.74 (-6.22; 0.68)	→			2016–2023	-4.24 (-9.16; -1.65)	↓		
			2020–2023	-0.29 (-2.82; 2.92)	→							
	Total	6.4					6.0					11.16 billion
Total	Male	5.0				-0.37 (-0.55; -0.21) ↓	2.6				-0.93 (-1.56; 0.00) →	30.71 billion
	Female	4.9				-0.32 (-0.54; -0.13) ↓	2.5				0.36 (-0.12; 1.01) →	**13.53 billion**
	Total	4.9				-0.35(-0.53; -0.19) ↓	2.6				-0.58 (-1.10; -0.04) ↓	44.24 billion

The proportion of in-hospital case fatality showed a decreasing trend between 2000 and 2023 (AAPC -0.58; 95%CI -1.10; -0.04) and remained stable by sex during the same period: men: AAPC 0.93; 95%CI -1.56; 0.00); women: AAPC 0.36; 95%CI -0.12; 1.01. The highest proportions were observed among older adults in both sexes (men: 7.2%; women: 5.0%). Deaths increased among girls aged 0–9 years between 2008 and 2016 (APC 4.96; 95%CI 2.60; 14.59) and among older women between 2013 and 2016 (APC 9.57; 95%CI 0.10; 13.56). Among men, an increase was observed in older adults from 2000 to 2021 (APC 0.79; 95%CI 0.45; 1.25) (Table 2). 

Hospitalizations generated a total cost of BRL 44.24 billion, representing 8.6% of the BRL 510.17 billion spent on all hospitalizations in the SUS. The mean annual cost was BRL 1.85 billion, and BRL 1,925.09 per approved hospitalization. The highest costs were incurred among young adults aged 20–39 years (BRL 15.48 billion). Men incurred higher costs than women across all age groups, except for older women, whose costs were higher than men’s. Men aged 20–39 years (BRL 12.49 billion) and older women (BRL 5.86 billion) accounted for the highest expenditures (Table 2).

By cause group, the highest mean hospital length of stay was observed for transport accidents and assaults (5.9 days each). Among men, this mean was higher than among women for all causes, except for falls (4.7 days). The mean length of stay for assaults showed a decreasing trend between 2000 and 2023 (AAPC -0.85; 95%CI -1.20; -0.56), with a period of increase from 2005 to 2012 among women (APC 2.62; 95%CI 1.43; 5.65). Hospitalizations due to falls maintained a stable mean length of stay (AAPC -0.05; 95%CI -0.23; 0.09), with periods of increase for both sexes: men, from 2005 to 2017 (APC 0.90; 95%CI 0.24; 2.14), and women, from 2007 to 2017 (APC 1.45; 95%CI 1.07; 2.46). The mean length of stay for self-inflicted injuries showed an increasing trend from 2000 to 2023 (AAPC 0.35; IC95% 0,12; 0,57). Among men, the increase occurred from 2019 to 2023 (APC 3.33; 95%CI 1.01; 7.69), whereas among women it occurred throughout the entire analyzed period, 2000 to 2023 (APC 0.36; 95%CI 0.03; 0.69) (Table 3). 

**Table 3:** Annual percent change (APC), average annual percent change (AAPC), 95% confidence intervals (95%CI) of in-hospital case fatality and mean length of stay, and total hospitalization costs for external causes funded by the Brazilian Unified Health System (SUS), by cause group and sex. Brazil, 2000–2023 (n=23,009,176)

Characteristics		Length of stay					Case fatality			Total costs (BRL)		
Cause groups	Sex	Mean^a^	Period	APV (95%CI)	Trend^b^	VMP (95%CI)	Case fatality (%)^c^	Period	APV (95%CI)	Trend ^b^	VMP (95%CI)	Adj. cost (BRL)^d^
**Transport accidents**	Male	6.0	2000–2017	-0.25 (0.48; 0.04)	→	0.82 (-1.07; -0.61) ↓	3.1	2000–2007	-0.19 (-1.24; 1.63)	→	-3.13 (-3.34; -2.86) ↓	7.67 billion
			2017–2023	-2.40 (-4.47; 1.44)	↓			2007–2010	-8.62 (-10.19; -4.87)	↓		
								2010–2023	-3.65 (-4.12; -2.13)	↓		
	Female	5.7	2000–2016	-0.35 (-0.63; 0.70)	→		2.8	2000–2007	1.44 (0.06; 3.64)	↑		2.06 billion
								2007–2010	-9.17 (-11.10; -4.62)	↓		
			2016–2023	-1.84 (-4.94; -0.92)	↓			2010–2023	-3.49 (-4.11; -1.35)	↓		
	Total	5.9					3.0					9.73 billion
Assaults	Male	6.0	2000–2017	-0.11(-0.40; 1.14)	→	-0.85 (-1.20; -0.56) ↓	5.2	2000–2015	-0.40 (-0.83; 0.19)	→	-1.32 (-1.68; -0.97) ↓	2.38 billion
								2015–2023	-3.04 (-4.96; -2.01)	↓		
			2017–2023	-2.04 (-6.16; -0.73)	↓							
	Female	5.2	2000–2005	-4.26 (-6.94; -2.48)	↓		3.6	2000–2012	2.53 (-2.15; 5.64)	→		394.66 million
			2005–2012	2.62 (1.43; 5.65)	↑			2012–2016	-10.84 (-17.05; 7.66)	→		
			2012–2023	-2.23 (-2.92; -1.65)	↓			2016–2023	-2.35 (-5.92; 8.02)	→		
	Total	5.9					4.9					2.77 billion
Falls	Male	4.4	2000–2005	-0.92 (-2.95; 0.11)	→	-0.05 (-0.23; 0.09) →	1.9	2000–2004	3.43 (1.78; 7.09)	↑	0.87 (0.63; 1.21) ↑	9.56 billion
			2005–2017	0.90 (0.24; 2.14)	↑			2004–2013	0.21 (-2.24; 0.68)	→		
			2017–2020	-3.26 (-4.33; 1.21)	→							
			2020–2023	-0.02 (-1.95; 2.36)	→			2013–2021	1.74 (1.21; 4.10)	↑		
								2021–2023	-4.97 (-8.61; -1.11)	↓		
	Female	4.7	2000–2007	-0.16 (-1.66; 0.45)	→		2.0	2000–2002	-1.47 (-3.74; 2.55)	→		5.93 million
			2007–2017	1.45 (1.07; 2.46)	↑			2002–2016	3.07 (2.79; 5.30)	↑		
			2017–2020	-4.39 (-5.50; -2.20)	↓							
			2020–2023	0.53 (-1.25; 3.33)	→			2016–2021	0.19 (-0.81; 2.59)	→		
								2021–2023	-6.67 (-9.97; -2.59)	↓		
	Total	4.5					1.9					15.49 billion
Self-inflicted injuries	Male	4.5	2000–2019	0.21 (-0.29; 0.44)	→	0.35 (0.12; 0.57) ↑	4.1	2000–2017	-0.17 (-5.02; 1.17)	→	0.81 (-0.19; 1.78) →	199.04 million
								2017–2023	7.78 (1.89; 26.69)	↑		
			2019–2023	3.33 (1.01; 7.69)	↑							
	Female	3.9	2000–2023	0.36 (0.03; 0.69)	↑		3.0	2000–2023	0.75 (-0.08; 1.61)	→		136.13 million
	Total	4.2					3.7					335.17 million
**Events of undetermined intent**	Male	5.2	2000–2004	-2.31 (-5.57; -0.52)	↓	-0.63 (-0.84; -0.38) ↓	2.6	2000–2009	2.38 (0.68; 6.70)	↑	0.01 (-0.59; 0.55) →	2.10 billion
			2004–2012	2.31 (1.62; 3.98)	↑			2009–2023	-1.52 (-3.19; -0.67)	↓		
			2012–2023	-2.30 (-2.75; -1.87)	↓							
	Female	5.2	2000–2004	-4.63 (-11.35; -1.04)	↓		2.8	2000–2016	3.46 (2.50; 4.70)	↑		944.11 million
			2004–2012	4.27 (2.94; 8.17)	↑			2016–2023	-4.40 (-9.20; -1.69)	↓		
			2012–2023	-2.57 (-3.47; -1.75)	↓							
	Total	5.2					2.6					3.04 billion
**Other external causes**	Male	4.9	2000–2004	-1.61 (-4.38; -0.08)	↓	-0.43 (-0.62; -0.25) ↓	2.7	2000–2013	-0.98 (-5.14; 0.33)	→	0.45 (-0.43; 1.05) →	8.75 billion
			2004–2016	0.69 (0.34; 2.03)	↑			2013–2016	12.07 (2.22; 17.29)	↑		
			2016–2023	-2.11 (-3.08; -1.39)	↓			2016–2023	-2.98 (-10.49; -0.28)	↓		
	Female	4.8	2000–2004	-1.13 (-3.14; 0.03)	→		2.9	2000–2013	0.98 (-2.00; 2.27)	→		4.07 billion
			2004–2017	0.52 (0.35; 1.46)	↑			2013–2016	19.28 (6.30; 25.75)	↑		
			2017–2020	-4.19 (-5.26; -2.31)	↓			2016–2023	-4.31 (-10.82; -1.52)	↓		
			2020–2023	0.42 (-1.26; 3.07)	→							
	Total	4.8					2.8					12.82 billion
Total		-	-	-	-		-	-	-	-		44.24 billion

Mean length of stay in days; ^b^↑ increasing, ↓ decreasing, → stable; ^c^Deaths; ^d^Cost adjusted (Adj. cost) for inflation, according to the broad Extended National Consumer Price Index (IPCA).

The highest proportions of in-hospital case fatality were observed for assaults and self-inflicted injuries in both sexes. Proportions increased significantly from 2017 to 2023 for hospitalizations due to self-inflicted injuries among men (APC 7.78; 95%CI 1.89; 26.69). Among women, higher case fatality proportions compared with men were observed in hospitalizations due to falls (2.0 deaths/100 hospitalizations for external causes), events of undetermined intent (2.8 deaths/100 hospitalizations for external causes), and other external causes (2.9 deaths/100 hospitalizations for external causes). For hospitalizations due to falls, an increasing trend in case fatality was observed from 2000 to 2023 (AAPC 0.87; 95%CI 0.63; 1.05), with periods of increase for both sexes. Hospitalizations due to falls and other external causes incurred the highest expenditures for both sexes, whereas self-inflicted injuries generated the lowest costs (Table 3). 

**Figure 2:**
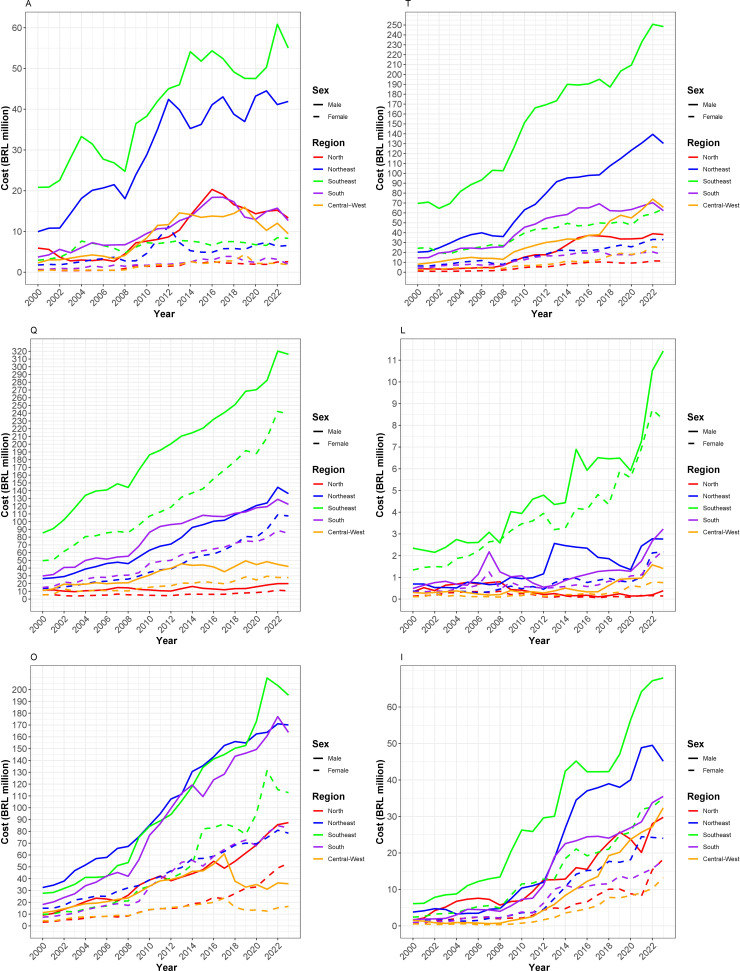
Hospitalization costs for assaults (A), transport accidents (T), falls (F), self-inflicted injuries (S), other external causes (O), and events of undetermined intent (U), funded by the Unified Health System, by region and sex. Brazil, 2000–2023 (n=23,009,176)

The highest hospitalization costs occurred in the Southeast (BRL 18.33 billion) and Northeast (BRL 10.26 billion). The male sex accounted for the highest costs (BRL 30.65 billion) across all regions and cause groups. The Southeast region presented the highest costs for both sexes in hospitalizations due to falls and self-inflicted injuries. In this region, the cost for women for these causes (BRL 3.06 billion for falls and BRL 90.35 million for self-inflicted injuries) exceeded the cost of hospitalizations among men for the same causes in the other regions (Figure 2).

## Discussion 

Hospitalizations due to external causes increased from 2000 to 2023 and were predominant among men and young adults. Falls, other external causes, and transport accidents were the most frequent causes. Mean length of stay and in-hospital case fatality increased with age. For both sexes, hospitalizations due to transport accidents and falls had the highest mean lengths of stay and the highest costs. In contrast, hospitalizations due to assaults and self-inflicted injuries had the highest case fatality and the lowest costs. 

This study presented some limitations. Coding errors in the International Statistical Classification of Diseases and Related Health Problems 10th Revision (ICD-10) may have reduced the total number of hospitalizations due to external causes. Incomplete and inconsistent variables in the Hospital Information System of the Unified Health System of the Brazilian Unified Health System (SIH/SUS) may have been a limiting factor. Accordingly, variables with low completion rates and hospital admission authorizations without a primary diagnosis were excluded from the study. The COVID-19 pandemic may also have affected the occurrence and recording of hospitalizations due to external causes.

Readmissions for the same individual or transfers to other health institutions cannot be identified in the SIH, which can create duplicate records and potentially affect the indicators. Hospitalization costs estimated from SIH records may also not reflect the actual financial situation of care, whether due to arbitration for particular interests, a lack of technical analysis, or poor public management, which could have led to under- or overestimation of public expenditures. 

The results of this study are consistent with other national and international studies (5,20,21), suggesting patterns of occurrence associated with sex, age, behaviors, and lifestyle, as well as inequalities in outcomes, service utilization, and associated costs (21). Men presented higher mean lengths of stay for transport accidents, reflecting the severity of injuries, possibly influenced by hegemonic masculinity behaviors (22). Motor vehicles may symbolize power and male superiority, as evidenced by excessive speed and negligent use of protective equipment, which, when combined with poorly maintained roads, increases the vulnerability of pedestrians, cyclists, and motorcyclists (5). 

The use of motorcycles as the primary mode of transportation among low-income families, the expansion of informal employment among young people, and the practice of transporting passengers without helmets exacerbate the situation, particularly in socially vulnerable contexts (11). Most survivors of these injuries face traumatic injuries, functional limitations, and impaired quality of life (11), with high hospitalization costs due to longer lengths of stay and the need for complex procedures (23), significantly impacting the health and social security systems. 

Hospitalizations due to assaults showed higher mean lengths of stay and higher case fatality proportions, reflecting the severity and complexity of required care, especially in cases with multiple injuries, and revealing a context of social vulnerability and persistence of interpersonal violence (22). Among women, although a decreasing trend in hospital length of stay was observed in some periods, case fatality remained stable throughout, suggesting underreporting or specificity in the violence experienced by women, predominantly domestic in nature and with different injury patterns (24). This situation calls for strengthening public policies addressing violence against women. 

The highest proportions of case fatality were due to self-inflicted injuries, especially among men, whose trend increased from 2017 to 2023. Underestimation of psychological suffering, stigma in seeking mental health care, rigid gender roles, financial difficulties, family conflicts, alcohol abuse, and hormonal disorders may contribute to this scenario (25). 

The literature highlights the “gender paradox” in suicide: women attempt suicide more frequently, while men experience more lethal outcomes, due to using more violent methods and exhibiting higher suicidal intent (25). Among women, lower case fatality from self-inflicted injuries may be associated with more consolidated support networks, greater attention to personal health, and use of less lethal means (26). This pattern shapes hospitalization rates and mean lengths of stay. Although these hospitalizations have lower aggregate costs than other causes, they have a significant impact and require multiprofessional and intersectoral support. 

The shortest mean hospital stays were observed among women, except for falls-related hospitalizations, particularly among older women. These results differ from those of other studies, in which the mean length of stay and case-fatality rates were higher among men (27,28). Females showed greater vulnerability in older age, while males predominated in active age groups, highlighting the importance of sex- and age-specific prevention strategies. 

Women represent the largest portion of Brazil’s elderly population and are more prone to falls (23) due to senescent decline (29), neurocognitive dysfunctions (27), polypharmacy (27), exposure to violence (7), inadequate urban and/or housing infrastructure (27), and limited access to appropriate care (27). The severity of these events may require complex interventions, resulting in substantial, unplanned expenditures for health services (23). Hospitalization costs in this group exceeded those of older men, underscoring the need for targeted policies for fall prevention and mental health care. These findings reflect Brazil’s epidemiological and demographic transition, characterized by a simultaneous, multifaceted burden of diseases arising from structural inequalities that affect all life stages (21).

The Southeast and Northeast regions had higher costs and hospitalization rates, reflecting their high population density (70% of Brazil’s population) (12). Urbanization, social inequalities, and the greater number of SUS-funded health institutions authorized for hospital care contribute to increased service utilization and reporting of these cases (21). 

Higher costs of hospitalizations due to external causes in the Southeast are attributable to large metropolitan areas with high rates of violence and accidents (21). In the Northeast, the high hospitalization costs may reflect socioeconomic vulnerabilities and the recent expansion of SUS coverage (21). That the most populous regions exhibited higher costs does not necessarily indicate lower risk in other regions, which may be characterized by underreporting and limited access to specialized health services, particularly in less developed areas. 

This study reinforces the use of the SIH as a tool for monitoring hospital morbidity and mortality and for planning health actions. It enabled an understanding of the distribution of hospitalizations due to external causes, their temporal trends, and associated costs, which remain a serious public health problem in Brazil. Despite reductions in case fatality, costs remain high, requiring strengthening of the health care network and control of the determinants of these health conditions. The findings also highlight the need to enhance the SIH further to enable continuous and effective monitoring.
