# Supplementary material for: Associated costs of hospitalizations due to external causes: time series analysis, Brazil, 2000-2023
Source: Epidemiol Serv Saude. 2026 Apr 10;35:e20240889. doi: 10.1590/S2237-96222026v35e20240889.en (PMC13073079; doi:10.1590/S2237-96222026v35e20240889.en)
Supplement: Supplementary file 1 [file 2237-9622-ress-35-e20240889-supp01-en.pdf]

Supplementary Figure 1. Completeness (%) and consistency (%) of variables from the Brazilian Unified Health System (*Sistema de Informações Hospitalares do SUS,* SIH/SUS), according to data completeness. Brazil, 2000–2023 (n=23,009,176)

Variable	Description	Completeness (%)	Consistency (%)
MUNIC_RES	Patient's place of residence, as reported by the hospital unit	100	100
UF_ZI	State to which the hospital unit is linked	100	100
ESPEC	Hospital bed specialty	100	100
CAR_INT	Type of hospitalization	100	100
CNES	National Registry of Health Establishments (*Cadastro Nacional de Estabelecimento de Saúde*, CNES) code of the institution	87.6	85.3
UTI_MES_TO	Total number of ICU days during hospitalization	100	100
DIAG_PRINC	Cause of hospitalization, according to the International Statistical Classification of Diseases and Related Health Problems 10th Revision (ICD-10)	100	100
DIAG_SECUN	Secondary diagnosis code, according to ICD-10	42.3	68.6
CID_ASSO	ICD code of the cause	39.5	38.1
CID_MORTE	ICD code of the cause of death	40.3	38.9
CBOR	Patient occupation, according to the Brazilian Classification of Occupations (*Classificação Brasileira de Ocupações*, CBO)	73.2	72.2
SEXO	Patient sex	100	100
IDADE	Age group	100	100
COD_IDADE	Unit of measurement of age	100	100
VAL_TOT	Amount related to approved (paid) Hospital Admission Authorizations (*Autorizações de Internação Hospitalar*, AIH) in the period	100	100
VAL_SH	Amount for hospital services	100	100
VAL_SP	Amount for professional services (PS) related to approved Hospital Admission Authorizations (AIH) in the period	100	100
QT_DIARIAS	Patient hospital bed-days	68.2	65.2
DIAS_PERM	Total hospitalization bed-days for approved/paid AIH during the period	100	100
DT_INTER	Admission date, in yyyymmdd format	100	100
DT_SAIDA	Discharge date, in yyyymmdd format	100	100
INSTRU	Patient education level	100	100
MORTE	Indicates if the patient died during hospitalization	100	100
RACA_COR	Patient race/skin color	68.2	73.5
IDENT	AIH identification	100	100
