# Supplementary material for: Associated costs of hospitalizations due to external causes: time series analysis, Brazil, 2000-2023
Source: Epidemiol Serv Saude. 2026 Apr 10;35:e20240889. doi: 10.1590/S2237-96222026v35e20240889.en (PMC13073079; doi:10.1590/S2237-96222026v35e20240889.en)
Supplement: Supplementary file 2 [file 2237-9622-ress-35-e20240889-supp01-pt.pdf]

Figura suplementar 1. Completude (%) e consistência (%) de variáveis do Sistema de Informações Hospitalares (SIH) segundo o preenchimento. Brasil, 2000-2023 (n=23.009.176)

**Variável**	**Descrição**	**Completude (%)**	**Consistência (%)**
MUNIC_RES	Local de residência do paciente, conforme informado pela unidade hospitalar	100	100
UF_ZI	Unidade da federação a qual a unidade hospitalar está vinculada	100	100
ESPEC	Especialidade do leito	100	100
CAR_INT	Caráter da internação	100	100
CNES	Código do CNES da instituição	87,6	85,3
UTI_MES_TO	Total de dias de UTI durante a internação	100	100
DIAG_PRINC	Causa da internação, segundo a Classificação Internacional das Doenças (CID-10)	100	100
DIAG_SECUN	Código do diagnóstico secundário, segundo o CID-10	42,3	68,6
CID_ASSO	CID da causa	39,5	38,1
CID_MORTE	CID da causa do óbito	40,3	38,9
CBOR	Ocupação do paciente, segundo a tabela da Classificação Brasileira de Ocupações	73,2	72,2
SEXO	Sexo do paciente	100	100
IDADE	Faixa etária	100	100
COD_IDADE	Unidade de medida da idade	100	100
VAL_TOT	Valor referente às AIH aprovadas (pagas) no período	100	100
VAL_SH	Valor serviços hospitalares	100	100
VAL_SP	Valor dos serviços profissionais (SP) referentes às autorizações de internações hospitalares (AIH) aprovadas no período.	100	100
QT_DIARIAS	Quantidade de diárias do paciente	68,2	65,2
DIAS_PERM	Total de dias de internação referentes às AIH aprovadas/pagas no período	100	100
DT_INTER	Data de internação, no formato aaaammdd	100	100
DT_SAIDA	Data de saída, no formato aaaammdd	100	100
INSTRU	Grau de instrução do paciente	100	100
MORTE	Indica se o paciente teve saída por óbito	100	100
RACA_COR	Raça/cor da pele do paciente	68,2	73,5
IDENT	Identificação da AIH	100	100
