# Supplementary material for: Associated costs of hospitalizations due to external causes: time series analysis, Brazil, 2000-2023
Source: Epidemiol Serv Saude. 2026 Apr 10;35:e20240889. doi: 10.1590/S2237-96222026v35e20240889.en (PMC13073079; doi:10.1590/S2237-96222026v35e20240889.en)
Supplement: Translation

# Translation: Custos associados das internações por causas externas: análise de série temporal, Brasil, 2000-2023

**DOI:** 10.1590/S2237-96222026v35e20240889.pt

**Authors:** Araceli Moreira De Martini Fontenele, Alcione Miranda dos Santos, Luz Marina Gómez Gómez, Fábio Nogueira da Silva, Bruno Luciano Carneiro Alves de Oliveira

**Affiliations:** 1 Universidade Federal do Maranhão, Departamento de Saúde Pública, São Luís, MA, Brasil

**Keywords:** Acidentes, Violência, Morbidade, Custos Hospitalares, Estudos de Séries Temporais

## Abstract

**Objetivo:**

Analisar a tendência temporal de letalidade e a média de permanência hospitalar, além de custos de internações por causas externas no Brasil entre 2000 e 2023.

**Métodos:**

Estudo de série temporal com dados de internações por causas externas registradas no Sistema de Informações Hospitalares. A análise de tendência temporal de média de permanência e de letalidade foi realizada por regressão por pontos de inflexão, com cálculo da variação percentual anual (VPA), da variação média no período (VMP) e intervalos de confiança de 95% (IC95%). Custos foram mensurados por macrocusteio.

**Resultados:**

De 2000 a 2023, foram registradas 23.009.176 (8,1%) internações por causas externas. A maioria no Sudeste (40,2%), entre homens de 20-39 anos (40,4%) e mulheres idosas (31,4%). Quedas foram o principal motivo (39,8%). Nesse período, a média de permanência foi decrescente em todas as faixas etárias, houve maior redução na faixa de 0-9 anos (VMP -1,27; IC95% -1,43; -1,13) e menor na de idosos (VMP -0,51; IC95% -0,77; -0,29). Houve tendência crescente na permanência de homens idosos entre 2003-2017 (VPA 0,41; IC95% 0,15; 2,46) e de mulheres idosas entre 2005-2017 (VPA 0,72; IC95% 0,30; 2,49). A letalidade apresentou tendência decrescente entre 2000-2023 (VMP -0,58; IC95% -1,10; -0,04), com maiores proporções em idosos (homens: 7,2%; mulheres: 5,0%). As internações geraram custo de R$ 44,24 bilhões, maiores gastos no Sudeste (R$ 18,33 bilhões) e entre homens (R$ 30,65 bilhões).

**Conclusão:**

Causas externas permanecem como grave problema de saúde do Brasil. Apesar de redução na letalidade, seus custos são elevados, demandam fortalecimento da rede de saúde e controle dos determinantes desses agravos.

**Aspectos éticos**

Esta pesquisa utilizou bancos de dados de domínio público e anonimizados:

## Introdução 

Causas externas estão entre os principais problemas de saúde pública global e as mais importantes causas de mortes prematuras e evitáveis, o que as torna responsáveis por quase 5 milhões (9%) de óbitos anuais no mundo (1). No Brasil, constituem o quarto grupo de causas de morte, depois de doenças cardiovasculares, respiratórias e neoplasias, e afetam sobretudo homens e jovens (1,2). Decorrentes de múltiplas iniquidades, as causas externas contribuem para elevada carga de internações, altos custos assistenciais e previdenciários, incapacidades e perda de produtividade dos indivíduos acometidos (1,3).

Internações por causas externas custam 6% do produto interno bruto brasileiro (3), com tendência crescente ao longo dos anos. Em 1998, a taxa de internação nos hospitais públicos conveniados ao Sistema Único de Saúde era de 37,1 casos/10 mil habitantes. Em 2010, a taxa passou para 48,7/10 mil habitantes (aumentou 31,3%). Em 2021, a taxa alcançou 58,4/10 mil habitantes: aumentou quase 20% na última década (4,5). No mesmo ano, quase 1,25 milhão de pessoas foram internadas no Brasil por causas externas, com tempo médio de internação de 4,7 dias e custo total (R$ 1,65 bilhão) seis vezes maior do que o de 2011 (1).

Esses agravos têm impactos em todo o sistema de saúde, já que geram elevado número de casos complexos que se somam aos decorrentes dos demais agravos à saúde, o que implica na reorganização de serviços, de recursos humanos e financeiros e de equipamentos (6). Apesar de haver mais análises dos dados de mortalidade, estima-se que, para cada óbito por lesão, existam 30 vítimas hospitalizadas por causas externas, o que sugere diversidade no perfil de morbidade em relação ao de mortalidade (7).

Estudos relatam aumento da incidência de agravos por causas externas (8,9). Mesmo assim, há pouca compreensão da distribuição, da tendência e das implicações socioeconômicas dessas internações. As causas externas apresentam heterogênea distribuição por características individuais e contextuais. Evidências apontam que características dos casos de internação estão associadas à mortalidade hospitalar, e que a assistência ofertada também determina o tempo de internação (10,11). Conhecer as características epidemiológicas da morbidade por causas externas pode subsidiar o planejamento de ações de vigilância em saúde e o monitoramento de políticas públicas. 

Este estudo teve por objetivo analisar a tendência temporal de letalidade e a média de permanência hospitalar, além dos custos de internações por causas externas no Brasil entre 2000 e 2023. 

## Métodos 

### Delineamento do estudo

Trata-se de análise de série temporal com dados de internações por causas externas registradas no Sistema de Informações Hospitalares (SIH) do Sistema Único de Saúde (SUS), custodiado pelo Departamento de Informação e Informática do SUS (DATASUS). Foram selecionados registros de autorizações de internações hospitalares (AIH) ocorridos entre 2000 a 2023.

### Contexto 

O Brasil, localizado na América do Sul, possui área de 8.510.417,77 km^2^ e população estimada em 203 milhões de habitantes. O país, composto por 27 unidades federativas (26 estados e um Distrito Federal), distribuídas em cinco regiões: Norte, Nordeste, Sudeste, Centro-Oeste e Sul, enfrenta desafios na saúde relacionados às internações por causas externas , que representam um problema de elevada magnitude e de alto impacto socioeconômico. As internações por esses agravos revelam sua dinâmica e seus custos associados (12).

### Participantes 

Existem duas abordagens para a obtenção de estimativas com o DATASUS e a classificação das causas externas: conservadora e não conservadora (12). Na conservadora, as internações são identificadas de duas maneiras: (1) quando, no diagnóstico principal, constam códigos (V01-Y98) do capítulo XX da Classificação Internacional de Doenças na sua 10ª revisão (CID-10) e qualquer outro código no diagnóstico secundário; (2) quando, no diagnóstico secundário, constam os códigos do capítulo XX da CID-10, o diagnóstico primário deve conter apenas os códigos do capítulo XIX da CID-10 (S00-T98), que se referem a lesões, envenenamentos e algumas outras consequências de causas externas (13). Na abordagem não conservadora, a contagem das internações é a partir da identificação do código da CID-10, esteja ele no diagnóstico principal ou no secundário. Na linha da base de dados do SIH e na coluna variável de diagnóstico principal ou diagnóstico secundário, deverá constar um código referente ao capítulo XX (13). 

Neste estudo, os registros de AIH foram selecionados segundo o ano de internação e identificados com a abordagem não conservadora (13). Essa abordagem permitiu obter mais casos em relação à abordagem conservadora e melhor observância da magnitude do fenômeno enquanto causa primária de internação. Na abordagem conservadora, com a obrigatoriedade da presença do capítulo XIX da CID-10 no diagnóstico principal, existe a possibilidade de alguns casos não serem contabilizados, o que não ocorre na abordagem não conservadora. Considerou-se também a possibilidade de identificação dos registros que tivessem sido codificados em desacordo com a CID-10 ou com base no SIH-SUS, mas que se tratassem de internações por causas externas (14,15). 

### Variáveis 

Foram utilizadas as seguintes variáveis: (i) sociodemográficas: faixa etária (em anos): 0-9; 10-19; 20-39; 40-59; ≥60); sexo (masculino; feminino); região do Brasil (Norte; Nordeste; Centro-Oeste; Sudeste; Sul); localização (capital; interior); (ii) grupos de causas externas: acidentes de transporte: V01-V99; agressões: X85-Y09; quedas: W00-W19; lesões autoprovocadas voluntariamente: X60-X84; eventos de intenção indeterminada: Y10-Y34; outras causas externas: W20-X59; (iii) indicadores: proporção de internação por causas externas; taxa de internações por causas externas pagas pelo SUS; média de permanência (em dias); proporção de letalidade hospitalar (óbitos); valor total das internações hospitalares pagas (custos em reais).

### **Fonte de dados**/mensuração 

A fonte de dados foi a base de AIH do SIH-SUS (base RD-AIH reduzida), custodiada pelo DATASUS. Foram empregados microdados não identificados captados pelo pacote microdatasus no software R (16). Inicialmente, foi instalado o pacote devtools no R. Em seguida, instalou-se o pacote microdatasus (repositório Github: https://github.com/) utilizando-se o código: devtools::install_github (“rfsaldanha/microdatasus”). Por fim, utilizou-se a função fetch_datasus para acesso, download e leitura dos microdados (16). 

A extração dos dados ocorreu por ano (2000 a 2023). A partir de 2014, houve modificação da estrutura da base de dados do SIH com inclusão de mais nove variáveis para registro de diagnósticos secundários: DIAGSEC1 a DIAGSEC9 (13). Os campos anteriormente designados para preenchimento de tal variável passaram a ser preenchidos por zeros. Para se obter os registros de internação sem prejuízos, foram criadas duas bases de dados.

Neste estudo, para analisar o preenchimento das variáveis (Tabela suplementar 1), utilizaram-se critérios de completude, consistência e confiabilidade (17). A completude foi classificada em: boa (≥75,1%); regular (50,1% a 75,0%); baixa (25,1% a 50,0%); muito baixa (≤25,0%). A consistência foi classifica em excelente (≥90%); regular (70% a 89%) e baixa (<70%). Variáveis com baixos percentuais de consistência não foram consideradas. Pela sua importância na identificação e caracterização das causas externas, apenas a variável diagnóstico secundário foi mantida, apesar de seu preenchimento deficiente já ter sido documentado. A confiabilidade baseou-se no cumprimento de critérios de completude (>50,1%) e de consistência (>70%). Após essa análise, as bases de dados foram agregadas. Utilizou-se base atualizada em 15/9/2024. Foram aplicados filtros: temporalidade de 2000 a 2023 e causas externas.

### Viés 

Os registros de AIH de longa permanência (AIH tipo 5: internações com duração >30 dias) foram excluídos da análise. Não foram incluídas causas externas decorrentes de causas específicas: complicações na assistência médica e cirúrgica (CID Y40-Y84); sequelas de causas externas de morbidade e de mortalidade (CID Y85-Y89); fatores suplementares relacionados a causas de morbimortalidade classificados em outra parte (CID Y90-Y98). Essa opção decorre do risco de duplicidade de registros: usuários poderiam ter internações anteriores pelas causas supracitadas e não representarem causas externas como evento primário que demandou a internação. 

### Métodos estatísticos

Inicialmente, realizou-se análise descritiva. Em seguida, estimaram-se quatro indicadores: (1) proporção de internação por causas externas (número de internações por causas externas segundo local de residência/número de internações por todas as causas x 100); (2) taxa de internação por causas externas pagas pelo SUS (número de internações por causas externas segundo local de residência/população residente x 100.000 habitantes); (3) média de permanência (total de dias de internação por causas externas/total de internações por causas externas no período); (4) proporção de letalidade hospitalar (internações por causas externas com saída por óbito/total de internações por causas externas no período x 100). O cálculo da proporção e da taxa de internação foi realizado por sexo. Para os demais indicadores, considerou-se sexo, faixa etária e grupo de causa externa. Os desfechos de internação foram alta e óbito.

No cálculo da taxa de internação, foram utilizados dados censitários e estimativas populacionais de cada região a partir do censo demográfico de 2010 do Instituto Brasileiro de Geografia e Estatística (12), o que disponibilizado pelo DATASUS. 

A análise de tendência temporal foi realizada para os indicadores média de permanência e letalidade hospitalar segundo sexo, faixa etária e grupo de causas externas. Os dados do estudo foram importados para o software Joinpoint Regression Program, versão 5.2.2.0, para análise linear segmentada da série temporal (regressão por pontos de inflexão) (18).

O Joinpoint Regression Program estimou a variação percentual anual (VPA) e a variação média no período (VMP). Classificou-se a tendência temporal em estacionária (p-valor>0,05), crescente (p-valor<0,05 e coeficiente da regressão positivo) ou decrescente (p-valor<0,05 e coeficiente da regressão negativo). Para todas as análises de tendências temporais, considerou-se intervalo de confiança de 95% (IC95%) (18).

Utilizou-se o método de macrocusteio nas análises dos custos (19). Esse método considera o valor total das AIH por causas externas pagas no período analisado, o tempo de permanência hospitalar e as despesas oriundas de serviços hospitalares. Tem como base o Índice Nacional de Preços ao Consumidor Amplo e considera as taxas anuais de inflação e valores atualizados para o ano-base, 2023. 

O cálculo do macrocusteio representa o número total de internações e os valores totais despendidos pelo SUS no SIH (custo total) nos períodos de 2000 a 2023. O custo médio das internações foi calculado a partir da divisão do custo total de internações por causas externas pelo número de AIH aprovadas (pagas) no período para internações por essas causas. A média do custo total foi calculada com a divisão do custo total pelo número de anos do período analisado (24 anos: 2000 a 2023). Os custos mensurados referiram-se exclusivamente às internações agudas. Alta e óbito foram considerados desfechos de internação. 

As análises foram realizadas com o Software RStudio versão 2024.4.4.0 (R Foundation for Statistical Computing, Boston, Estados Unidos). 

## Resultados 

De 2000 a 2023, 283.149.255 internações hospitalares foram registradas no Sistema de Informações Hospitalares (SIH) do Sistema Único de Saúde (SUS). Dessas, 23.009.176 (8,1%) foram por causas externas. As internações predominaram nas idades entre 20-39 anos (35,6%) e entre 40-59 anos (24,1%), nas regiões Sudeste (40,2%) e Nordeste (24,7%) e em cidades fora das capitais (77,3%). Observaram-se diferenças relevantes para todas as variáveis avaliadas segundo o sexo dos pacientes. Nos homens, as internações predominaram entre 20-39 anos (40,4%) e 40-59 anos (24,5%); nas mulheres, elas foram maiores em idosas (≥60 anos) (31,4%). As internações foram mais frequentes em duas regiões para ambos os sexos: homens: 39,5% no Sudeste e 25,3% no Nordeste; mulheres: 41,7% e 23,4%. A queda permaneceu como a causa mais frequente (39,8%) dentro dos grupos de causas, seguida de outras causas externas (28,5%) (Tabela 1). 

**Tabela 1:** Frequências absolutas (n) e frequências relativas (%) dos casos de internações por causas externas financiadas pelo Sistema Único de Saúde segundo características sociodemográficas e grupos de causas, por sexo. Brasil, 2000-2023 (n=23.009.176)

Características	Total	Masculino	Feminino
	n (%)	n (%)	n (%)
**Faixa etária** (anos)			
0-9	2.127.047 (9,2)	1.344.092 (8,5)	782.955 (10,9)
10-19	3.012.101 (13,1)	2.314.262 (14,6)	697.839 (9,8)
20-39	8.182.711 (35,6)	6.408.019 (40,4)	1.774.692 (24,8)
40-59	5.534.824 (24,1)	3.881.163 (24,5)	1.653.661 (23,1)
≥60	4.152.493 (18,0)	1.906.937 (12,0)	2.245.556 (31,4)
**Região do país**			
Norte	2.098.646 (9,1)	1.503.814 (9,5)	594.832 (8,3)
Nordeste	5.688.721 (24,7)	4.013.906 (25,3)	1.674.815 (23,4)
Centro-Oeste	2.211.728 (9,6)	1.540.873 (9,7)	670.855 (9,4)
Sudeste	9.244.974 (40,2)	6.261.088 (39,5)	2.983.886 (41,7)
Sul	3.765.107 (16,4)	2.534.792 (16,0)	1.230.315 (17,2)
Localização			
Capital	5.212.116 (22,7)	3.538.352 (22,3)	1.673.764 (23,4)
Interior	17.797.060 (77,3)	12.316.121 (77,7)	5.480.939 (76,6)
**Grupos de causas**			
Acidentes de transporte	4.217.581 (18,4)	3.259.714 (20,6)	957.867 (13,4)
Agressões	1.153.999 (5,0)	954.786 (6,0)	199.213 (2,8)
Quedas	9.153.177 (39,8)	5.851.763 (36,9)	3.301.414 (46,1)
Lesões autoprovocadas voluntariamente	243.901 (1,0)	141.414 (0,9)	102.487 (1,4)
Eventos de causa indeterminada^a^	1.691.063 (7,3)	1.163.413 (7,3)	527.650 (7,4)
Outras causas externas^b^	6.549.455 (28,5)	4.483.383 (28,3)	2.066.072 (28,9)

^a^Eventos de causa indeterminada contemplam agravos sobre os quais a informação não permite fazer a distinção entre acidente, lesão autoprovocada e agressão; ^b^Outras causas externas contemplam exposição a forças mecânicas, afogamento e submersão acidental, outros riscos acidentais à respiração, queimaduras e envenenamentos (intoxicação acidental).

No período sob análise (2000-2023), a proporção de internação hospitalar por causas externas nos homens passou de 5,4% para 10,6%, aumento de 96,3%. Entre mulheres, cresceu 146,1%, de 2,6% para 6,4%. Já a taxa de internação por causas externas, nos homens aumentou 58,8%, de 386,5 para 613,7 internações/100 mil habitantes. Entre mulheres, o aumento foi de 84,3%, de 226,2 para 416,8/100 mil habitantes (Figura 1). 

**Figura 1:** Proporção (P) e taxa de internações (T) por causas externas financiadas pelo SUS segundo o sexo. Brasil, 2000-2023 (n=23.009.176)

Verificou-se tendência decrescente da média de permanência hospitalar em todas as faixas etárias. A maior redução foi entre 0-9 anos (VMP -1,27; IC95% -1,43; -1,13) e a menor entre idosos (VMP -0,51; IC95% -0,77; -0,29). As maiores médias de permanência hospitalar foram em idosos, 6,6 dias para homens e 6,3 para mulheres. Nos homens, observou-se tendência crescente entre adolescentes no período de 2004 a 2016 (VPA 0,80; IC95% 0,47; 1,63) e entre idosos de 2003 a 2017 (VPA 0,41; IC95% 0,15; 2,46). Nas mulheres, a tendência foi crescente em adultas na faixa etária 20-39 anos entre 2005 e 2013 (VPA 1,19; IC95% 0,57; 3,08) e na faixa 40-59 anos de 2004 a 2015 (VPA 1,65; IC95% 0,47; 8,10), bem como em idosas no período entre 2005 e 2017 (VPA 0,72; IC95% 0,30; 2,49) (Tabela 2). 

**Tabela 2:** Variação percentual anual (VPA), variação anual média no período (VMP), intervalos de confiança de 95% (IC95%) de letalidade hospitalar e média de permanência, e custos totais de internações hospitalares por causas externas financiadas pelo Sistema Único de Saúde, segundo faixa etária e sexo. Brasil, 2000-2023 (n=23.009.176)

Características		Permanência					Letalidade			Custos totais		
Faixa etária (anos)	Sexo	Média^a^	Período	VPA (IC95%)	Tendência^b^	VMP (IC95%)	Letalidade (%)^c^	Período	VPA (IC95%)	Tendência ^b^	VMP (IC95%)	Custo-a (R$)^d^
0-9	Masculino	3,4	2000-2006	-2,10 (-3,82; -1,39)	↓	-1,27 (-1,43; -1,13) ↓	0,6	2000-2023	-0,49 (-1,03; 0,06)	→	-0,30 (-0,92; 0,11) →	1,46 bilhão
			2006-2016	-0,41 (-0,74; 0,94)	→							
			2016-2023	-2,25 (-3,04; -1,73)	↓							
	Feminino	3,4	2000-2013	-1,17 (-1,76; -0,69)	↓		0,8	2000-2008	-2,01 (-10,21; 0,32)	→		885,57 milhões
			2013-2017	1,67 (-1,68; 3,54)	→			2008-2016	4,96 (2,60; 14,59)	↑		
			2017-2020	-5,27 (-6,76; 1,19)	→			2016-2023	-2,65 (-7,77; -0,27)	↓		
			2020-2023	0,03 (-2,68; 3,47)	→							
	Total	3,4					0,7					2,34 bilhões
10-19	Masculino	4,0	2000-2004	-1,95(-4,66; -0,33)	↓	-0,85 (-0,99; -0,72) ↓	1,3	2000-2017	-1,17 (-1,75; -0,48)	↓	-2,90 (-3,39; -2,51) ↓	3,48 bilhões
								2017-2023	-7,73 (-11,42; -5,44)	↓		
			2004-2016	0,80 (0,47; 1,63)	↑							
			2016-2023	-3,30 (-4,07; -2,61)	↓							
	Feminino	3,9	2000-2016	-0,12 (-0,70; 2,97)	→		1,0	2000-2010	-1,23 (-2,51; 3,35)	→		991,33 milhões
			2016-2023	-2,21 (-7,01; -0,50)	↓			2010-2023	-4,19 (-6,55; -3,34)	↓		
	Total	4,0					1,2					4,47 bilhões
20-39	Masculino	4,9	2000-2005	-1,01 (-3,14; -0,04)	↓		2,1	2000-2004	0,03 (-2,32; 5,70)	→		12,49 bilhões
			2005-2015	0,42 (-0,10; 1,84)	→			2004-2023	-3,41 (-3,88; -3,17)	↓		
			2015-2023	-2,13 (-2,74; -1,55)	↓							
	Feminino	4,3	2000-2005	-1,55 (-4,20; -0,40)	↓	-0,80 (-0,97; -0,64) ↓	1,3	2000-2005	1,14 (-1,73; 9,27)	→	-2,90 (-3,17; -2,58) ↓	2,99 bilhões
			2005-2013	1,19 (0,57; 3,08)	↑			2005-2023	-3,73 (-4,50; -3,30)	↓		
			2013-2023	-1,92 (-2,41; -1,48)	↓							
	Total	4,8					1,9					15,48 bilhões
40-59	Masculino	5,5	2000-2023	-0,41 (-0,99; 0,16)	→	-0,82 (-1,00; -0,63) ↓	2,8	2000-2023	-2,29 (-2,69; -1,91)	↓	-2,48 (-3,41; -1,11) ↓	7,93 bilhões
	Feminino	4,7	2000-2004	-5,46 (-13,70; -1,02)	↓		1,8	2000-2021	-0,66 (-1,29; 2,53)	→		2,87 bilhões
			2004-2015	1,65 (0,47; 8,10)	↑			2021-2023	-15,0 (-25,39; -1,53)	↓		
			2015-2023	-2,85 (-8,09;-0,94)	↓							
	Total	5,3					2,5					10,80 bilhões
≥60	Masculino	6,6	2000-2003	-1,83 (-4,18; 0,09)	→	-0,51 (-0,77; -0,29) ↓	7,2	2000-2021	0,79 (0,45; 1,25)	↑	-0,02 (-0,59; 0,73) →	5,30 bilhões
			2003-2017	0,41 (0,15; 2,46)	↑			2021-2023	-11,91 (-16,88; -3,17)	↓		
			2017-2020	-4,19 (-5,71; 0,35)	→							
			2020-2023	0,13 (-2,44; 3,39)	→							
	Feminino	6,3	2000-2005	-0,95 (-3,48; 0,17)	→		5,0	2000-2013	1,52 (-3,43; 2,85)	→		5,86 bilhões
			2005-2017	0,72 (0,30; 2,49)	↑			2013-2016	9,57 (0,10; 13,56)	↑		
			2017-2020	-4,74 (-6,22; 0,68)	→			2016-2023	-4,24 (-9,16; -1,65)	↓		
			2020-2023	-0,29 (-2,82; 2,92)	→							
	Total	6,4					6,0					11,16 bilhões
Total	Masculino	5,0				-0,37 (-0,55; -0,21) ↓	2,6				-0,93 (-1,56; 0,00) →	30,71 bilhões
	Feminino	4,9				-0,32 (-0,54; -0,13) ↓	2,5				0,36 (-0,12; 1,01) →	13,53 bilhões
	Total	4,9				-0,35(-0,53; -0,19) ↓	2,6				-0,58 (-1,10; -0,04) ↓	44,24 bilhões

^a^média de permanência em dias; ^b^↑ crescente, ↓ decrescente, → estacionária; ^c^óbitos; ^d^custo ajustado (custo-^a^) pela inﬂação, segundo o índice nacional de preços ao consumidor amplo.

A proporção de letalidade teve tendência decrescente entre 2000 e 2023 (VMP -0,58; IC95% -1,10; -0,04) e foi estável por sexo no mesmo período: homens: VMP -0,93; IC95% -1,56; 0,00; mulheres: VMP 0,36; IC95% -0,12; 1,01. As maiores proporções foram entre idosos em ambos os sexos (homens: 7,2%; mulheres: 5,0%). Os óbitos aumentaram entre mulheres na faixa etária 0-9 anos entre 2008 e 2016 (VPA 4,96; IC95% 2,60; 14,59) e em idosos entre 2013 e 2016 (VPA 9,57; IC95% 0,10; 13,56). Entre homens, o aumento foi observado em idosos no período de 2000 a 2021 (VPA 0,79; IC95% 0,45; 1,25) (Tabela 2). 

As internações geraram custo total de R$ 44.24 bilhões: 8,6% dos R$ 510,17 bilhões que foram gastos em todas as internações no SUS. O custo médio anual foi de R$ 1,85 bilhão, e de R$ 1.925,09 por autorização de internação hospitalar. Os maiores custos foram em adultos jovens entre 20-39 anos (R$ 15,48 bilhões). Homens apresentaram maiores custos em todas as faixas etárias em relação a mulheres, exceto quanto às idosas, em que os custos foram maiores. Homens de 20-39 anos (R$ 12,49 bilhões) e mulheres idosas (R$ 5,86 bilhões) apresentaram os maiores gastos (Tabela 2).

Por grupo de causa, a maior média de permanência hospitalar foi por acidentes de transporte e por agressões (5,9 dias para cada). Entre homens, essa média foi maior do que entre mulheres para todas as causas, com exceção das quedas (4,7 dias). A média de permanência por agressões teve tendência decrescente entre 2000 e 2023 (VMP 0,85; IC95% _-_1,20; -0,56), com períodos de aumento de 2005 a 2012 entre mulheres (VPA 2,62; IC95% 1,43; 5,65). As internações por queda mantiveram média de permanência estável (VMP -0,05; IC95% -0,23; 0,09), com períodos de aumento para ambos os sexos: homens, de 2005 a 2017 (VPA 0,90; IC95% 0,24; 2,14), e mulheres, de 2007 a 2017 (VPA 1,45; IC95% 1,07; 2,46). A média de permanência por lesões autoprovocadas apresentou tendência crescente de 2000 a 2023 (VMP 0,35; IC95% 0,12; 0,57). Nos homens, o aumento ocorreu de 2019 a 2023 (VPA 3,33; IC95% 1,01; 7,69), enquanto nas mulheres, foi em todo o período em análise, de 2000 a 2023 (VPA 0,36; IC95% 0,03; 0,69) (Tabela 3). 

**Tabela 3:** Variação percentual anual (VPA), variação anual média no período (VMP), intervalos de confiança de 95% (IC95%) de letalidade hospitalar e média de permanência e custos totais de internações hospitalares por causas externas financiadas pelo Sistema Único de Saúde, segundo grupo de causas e sexo. Brasil, 2000-2023 (n=23.009.176)

Características		Permanência					Letalidade			Custos totais		
Grupo de causas	Sexo	Média^a^	Período	VPA (IC95%)	Tendência^b^	VMP (IC95%)	Letalidade (%)^c^	Período	VPA (IC95%)	Tendência ^b^	VMP (IC95%)	Custo-a (R$)^d^
**Acidentes de transporte**	Masculino	6,0	2000 -2017	-0,25 (0,48; 0,04)	→	0,82 (-1,07; -0,61) ↓	3,1	2000-2007	-0,19 (-1,24; 1,63)	→	-3,13 (-3,34; -2,86) ↓	7,67 bilhões
			2017-2023	-2,40 (-4,47; 1,44)	↓			2007-2010	-8,62 (-10,19; -4,87)	↓		
								2010-2023	-3,65 (-4,12; -2,13)	↓		
	Feminino	5,7	2000-2016	-0,35 (-0,63; 0,70)	→		2,8	2000-2007	1,44 (0,06; 3,64)	↑		2,06 bilhões
								2007-2010	-9,17 (-11,10; -4,62)	↓		
			2016-2023	-1,84 (-4,94; -0,92)	↓			2010-2023	-3,49 (-4,11; -1,35)	↓		
	Total	5,9					3,0					9,73 bilhões
Agressões	Masculino	6,0	2000-2017	-0,11(-0,40; 1,14)	→	-0,85 (-1,20; -0,56) ↓	5,2	2000-2015	-0,40 (-0,83; 0,19)	→	-1,32 (-1,68; -0,97) ↓	2,38 bilhões
								2015-2023	-3,04 (-4,96; -2,01)	↓		
			2017-2023	-2,04 (-6,16; -0,73)	↓							
	Feminino	5,2	2000-2005	-4,26 (-6,94; -2,48)	↓		3,6	2000-2012	2,53 (-2,15; 5,64)	→		394,66 milhões
			2005-2012	2,62 (1,43; 5,65)	↑			2012-2016	-10,84 (-17,05; 7,66)	→		
			2012-2023	-2,23 (-2,92; -1,65)	↓			2016-2023	-2,35 ( -5,92; 8,02)	→		
	Total	5,9					4,9					2,77 bilhões
Quedas	Masculino	4,4	2000-2005	-0,92 (-2,95; 0,11)	→	-0,05 (-0,23; 0,09) →	1,9	2000-2004	3,43 (1,78; 7,09)	↑	0,87 (0,63; 1,05) ↑	9,56 bilhões
			2005-2017	0,90 (0,24; 2,14)	↑			2004-2013	0,21 ( -2,24; 0,68)	→		
			2017-2020	-3,26 (-4,33; 1,21)	→							
			2020-2023	-0,02 (-1,95; 2,36)	→			2013-2021	1,74 (1,21; 4,10)	↑		
								2021-2023	-4,97 (-8,61; -1,11)	↓		
	Feminino	4,7	2000-2007	-0,16 (-1,66; 0,45)	→		2,0	2000-2002	-1,47 (-3,74; 2,55)	→		5,93 milhões
			2007-2017	1,45 (1,07; 2,46)	↑			2002-2016	3,07 (2,79; 5,30)	↑		
			2017-2020	-4,39 (-5,50; -2,20)	↓							
			2020-2023	0,53 (-1,25; 3,33)	→			2016-2021	0,19 (-0,81; 2,59)	→		
								2021-2023	-6,67 (-9,97; -2,59)	↓		
	Total	4,5					1,9					15,49 bilhões
**Lesões auto** provocadas	Masculino	4,5	2000-2019	0,21 (-0,29; 0,44)	→	0,35 (0,12; 0,57) ↑	4,1	2000-2017	-0,17 (-5,02; 1,17)	→	0,81 (-0,19; 1,78) →	199,04 milhões
								2017-2023	7,78 (1,89; 26,69)	↑		
			2019-2023	3,33 (1,01; 7,69)	↑							
	Feminino	3,9	2000-2023	0,36 (0,03; 0,69)	↑		3,0	2000-2023	0,75 (-0,08; 1,61)	→		136,13 milhões
	Total	4,2					3,7					335,17 milhões
**Eventos de causa indeterminada**	Masculino	5,2	2000-2004	-2,31 (-5,57; -0,52)	↓	-0,63 (-0,84; -0,38) ↓	2,6	2000-2009	2,38 (0,68; 6,70)	↑	0,01 (-0,59; 0,55) →	2,10 bilhões
			2004-2012	2,31 (1,62; 3,98)	↑			2009-2023	-1,52 (-3,19; -0,67)	↓		
			2012-2023	-2,30 (-2,75; -1,87)	↓							
	Feminino	5,2	2000-2004	-4,63 (-11,35; -1,04)	↓		2,8	2000-2016	3,46 (2,50; 4,70)	↑		944,11 milhões
			2004-2012	4,27 (2,94; 8,17)	↑			2016-2023	-4,40 (-9,20; -1,69)	↓		
			2012-2023	-2,57 (-3,47; -1,75)	↓							
	Total	5,2					2,6					3.04 bilhões
**Outras causas externas**	Masculino	4,9	2000-2004	-1,61 (-4,38; -0,08)	↓	-0,43 (-0,62; -0,25) ↓	2,7	2000-2013	-0,98 (-5,14; 0,33)	→	0,45 (-0,43; 1,05) →	8,75 bilhões
			2004-2016	0,69 (0,34; 2,03)	↑			2013-2016	12,07 (2,22; 17,29)	↑		
			2016-2023	-2,11 (-3,08; -1,39)	↓			2016-2023	-2,98 (-10,49; -0,28)	↓		
	Feminin	4,8	2000-2004	-1,13 (-3,14; 0,03)	→		2,9	2000-2013	0,98 (-2,00; 2,27)	→		4,07 bilhões
			2004-2017	0,52 (0,35; 1,46)	↑			2013-2016	19,28 (6,30; 25,75)	↑		
			2017-2020	-4,19 (-5,26; -2,31)	↓			2016-2023	-4,31 (-10,82; -1,52)	↓		
			2020-2023	0,42 (-1,26; 3,07)	→							
	Total	4,8					2,8					12,82 bilhões
Total		-	-	-	-		-	-	-	-		44.24 bilhões

^a^média de permanência em dias; ^b^ ↑ crescente, ↓ decrescente, → estacionária; ^c^óbitos; ^d^custo ajustado (custo-a) pela inﬂação, segundo o índice nacional de preços ao consumidor amplo.

As maiores proporções de letalidade foram por agressões e por lesões autoprovocadas em ambos os sexos. As proporções aumentaram significativamente de 2017 a 2023 nas internações por lesões autoprovocadas entre homens (VPA 7,78; IC95% 1,89; 26,69). Entre mulheres, houve maior proporção de letalidade em relação a homens nas internações por quedas (2,0 óbitos/100 internações por causas externas), por eventos de intenção indeterminada (2,8 óbitos/100 internações por causas externas) e por outras causas externas (2,9 óbitos/100 internações por causas externas). Nas internações por quedas, observou-se tendência crescente da letalidade de 2000 a 2023 (VMP 0,87; IC95% 0,63; 1,05), com períodos de aumento para ambos os sexos. Internações por quedas e por outras causas externas demandaram maiores gastos para ambos os sexos, enquanto menores gastos foram para lesões autoprovocadas (Tabela 3). 

Os maiores custos de internações hospitalares ocorreram no Sudeste (R$ 18,33 bilhões) e no Nordeste (R$ 10,26 bilhões). O sexo masculino foi o responsável pelos maiores custos (R$ 30,65 bilhões) em todas as regiões e em todos os grupos de causas. O Sudeste apresentou maiores custos para ambos os sexos nas internações por quedas e por lesões autoprovocadas. Nessa região, o custo para o sexo feminino de internações por tais causas (R$ 3,06 bilhões em quedas e R$ 90,35 milhões em lesões autoprovocadas) superou o custo de internações de homens para essas mesmas causas nas demais regiões (Figura 2).

**Figura 2:** Custos de internações hospitalares por agressões (A), acidentes de transporte (T), quedas (Q), lesões autoprovocadas (L), outras causas externas (O) e eventos de intenção indeterminada (I) financiadas pelo Sistema Único de Saúde, segundo região e sexo. Brasil, 2000-2023 (n=23.009.176)

## Discussão 

Internações por causas externas aumentaram de 2000 a 2023 e foram predominantes em homens e adultos jovens. Quedas, outras causas externas e acidentes de transporte foram as causas mais frequentes. A média de permanência e a letalidade hospitalar aumentaram com a idade. Em ambos os sexos, internações por acidentes de transporte e quedas apresentaram as maiores médias de permanência hospitalar e os maiores custos, e internações por agressões e por lesões autoprovocadas, a maior letalidade e os menores custos. 

Este estudo apresentou algumas limitações. Erros de codificação na Classificação Internacional de Doenças - 10ª revisão (CID-10) podem ter reduzido o universo de internações por causas externas. A incompletude e a inconsistência de variáveis no Sistema de Informações Hospitalares (SIH) do Sistema Único de Saúde (SUS) podem ter sido um fator limitante. Quanto a isso, variáveis com baixo percentual de preenchimento e autorizações de internações hospitalares sem diagnóstico principal não foram incluídas no estudo. E a pandemia de covid-19 pode ter afetado a ocorrência e o registro de internações por causas externas.

As reinternações de um mesmo indivíduo ou a transferência para outra instituição de saúde não são passíveis de identificação no SIH, de forma que é possível duplicidade de registro e impactos nos indicadores. Custos de internações estimados com base nos registros do SIH também podem não ter refletido a real situação financeira dos atendimentos, seja por arbitragem por interesses particulares, pela falta de análise técnica ou por má gestão pública, o que pode ter gerado sub ou superfaturamento de contas públicas. 

Os resultados deste estudo são consistentes com outros estudos nacionais e internacionais (5,20,21), o que sugere padrões de ocorrência associados ao sexo, à idade, a comportamentos e hábitos de vida e a desigualdades nos desfechos, na utilização dos serviços e nos custos associados (21). Homens apresentaram maior média de permanência hospitalar por acidentes de transporte, o que refletiu a gravidade das lesões, possivelmente influenciadas por atitudes de masculinidade hegemônica (22). Veículos automotores podem representar poder e superioridade masculina, expressos no uso de velocidade excessiva e na negligência no uso de equipamentos de proteção, o que, somado à má conservação de vias, torna pedestres, ciclistas e motociclistas mais vulneráveis (5). 

O uso da motocicleta como principal meio de locomoção por famílias de baixa renda, a expansão do trabalho informal entre jovens e o transporte de passageiros sem capacete agravam a situação, principalmente em contextos de vulnerabilidade social (11). A maioria dos sobreviventes desses agravos enfrenta lesões traumáticas, limitações funcionais e comprometimento na qualidade de vida (11), com altos custos associados na internação pela necessidade de maior tempo de permanência e de procedimentos complexos (23), o que impacta significativamente o sistema de saúde e o previdenciário. 

As internações por agressões apresentaram maiores médias de permanência e maiores proporções de letalidade, o que evidenciou a gravidade e a complexidade do cuidado necessário, especialmente quando há múltiplas lesões, além de revelar um cenário de vulnerabilidade social e de persistência da violência interpessoal (22). Entre as mulheres, embora se observe tendência decrescente na permanência hospitalar em alguns períodos, a letalidade manteve-se estacionária durante todo o período, o que sugeriu subnotificação ou especificidade na violência sofrida por mulheres, majoritariamente de natureza doméstica e com diferentes padrões de lesão (24). Essa situação demanda fortalecimento de políticas públicas de enfrentamento à violência contra a mulher. 

As maiores proporções de letalidade foram por lesões autoprovocadas, especialmente entre homens, cuja tendência cresceu de 2017 a 2023. Subestimação do sofrimento psíquico, estigmas na busca por cuidado em saúde mental, rigidez nos papéis de gênero, dificuldades financeiras, conflitos familiares, abuso de álcool e desordens hormonais podem contribuir para esse cenário (25). 

A literatura destaca o “paradoxo de gênero” no suicídio: mulheres realizam mais tentativas e homens concentram desfechos letais, por utilizarem métodos mais violentos e apresentarem maior intenção suicida (25). Entre mulheres, menor letalidade por lesões autoprovocadas pode estar associada a redes de apoio mais consolidadas, a maior atenção à própria saúde e a uso de meios menos letais (26). Esse padrão modela taxas de internação e média de permanência. Apesar de menor custo agregado comparado a outras causas, essas internações possuem impacto expressivo e exigem suporte multiprofissional e intersetorial. 

As menores médias de permanência hospitalar foram entre mulheres, exceto nas internações por quedas, especialmente em mulheres idosas. Esses resultados diferem de outros estudos, nos quais tempo de permanência hospitalar e proporção de letalidade foram maiores entre homens (27,28). O sexo feminino evidenciou maior vulnerabilidade na fase idosa, enquanto o masculino apresentou predominância em faixas etárias ativas, o que reforçou a importância de estratégias de prevenção específicas por sexo e por faixa etária. 

Mulheres constituem a maior parcela da população idosa do Brasil e estão mais propensas a quedas (23) devido ao declínio senescente (29), a disfunções neurocognitivas (27), a polifarmácia (27), a violência (7), a infraestrutura urbana e/ou habitacional inadequada (27) e a acesso limitado à assistência adequada (27). A gravidade desses eventos pode exigir intervenções complexas, o que gera gastos elevados e não planejados para os serviços de saúde (23). Custos de internação nesse grupo mostraram superação em relação aos homens idosos, o que evidenciou a necessidade de políticas específicas para prevenção de quedas e atenção em saúde mental. Esses achados refletiram a transição epidemiológica e demográfica brasileira, marcada pela carga múltipla e simultânea de doenças, decorrentes de desigualdades estruturais que impactam todas as fases da vida (21).

As regiões Sudeste e Nordeste apresentaram maiores custos e taxas de internações mais elevadas, o que revelou a alta densidade demográfica dessas regiões (70% da população brasileira) (12). Urbanização, desigualdades sociais e maior número de instituições de saúde financiadas pelo SUS e habilitadas para internação são fatores que contribuem para o aumento de atendimentos e para os registros desses casos (21). 

Maiores custos de internações por causas externas na região Sudeste devem-se à existência de grandes metrópoles com elevados índices de violência e de acidentes (21). No Nordeste, o alto custo de internações pode refletir vulnerabilidades socioeconômicas e aumento recente na cobertura do SUS (21). Que as regiões mais populosas tenham apresentado maiores custos não é forçoso indicativo de menor risco nas demais regiões, que podem ser caracterizadas por subnotificação e por acesso limitado a serviços de saúde especializados, principalmente nas áreas menos desenvolvidas. 

Este estudo reforçou a utilização do SIH como ferramenta de vigilância da morbimortalidade hospitalar e de planejamento de ações de saúde. Possibilitou compreender a distribuição das internações por causas externas, sua tendência temporal e seus custos associados , que permanecem como grave problema de saúde do Brasil. Apesar de redução da letalidade, seus custos ainda são elevados, demandam fortalecimento da rede de saúde e controle dos determinantes desses agravos. Os achados reforçam também a necessidade de maior qualificação do SIH para monitoramentos contínuos e efetivos.
